# Pollinator‐Mediated Interactions Affect Patterns of Selection on Floral Traits of Co‐Flowering Plants

**DOI:** 10.1002/ece3.71166

**Published:** 2025-03-16

**Authors:** Yan Ma, Xiaoli Wang, Yizhi Qiu, Zhigang Zhao

**Affiliations:** ^1^ State Key Laboratory of Herbage Improvement and Grassland Agroecosystems College of Ecology, Lanzhou University Lanzhou China

**Keywords:** dominant flowering species, floral traits, phenotypic selection, pollinator‐mediated interaction

## Abstract

The importance of species interactions in shaping the evolution of ecological communities is well established, as they can significantly alter biotic selection. Pollinator‐mediated plant–plant interactions on plant reproductive performance can vary from facilitation to competition. Although the richness and density of co‐flowering species influence patterns of selection, the role of key species in an ecological community remains unclear. We experimentally removed flowers of a dominant flowering species, *Ranunculus tanguticus*, in an alpine meadow on the Qinghai‐Tibet Plateau, and examined how this dominant affected fitness components and phenotypic selection on floral traits of five neighboring species via stigmatic pollen load. *R. tanguticus* had a positive effect on the pollen receipt of two plant species, *A. obtusiloba* and *A. souliei* . Correspondingly, flower attractive traits (flower height and size) rather than the mechanical‐fit trait (stigma position) of both plants were subjected to significant net selection (i.e., selection differential) when *R. tanguticus* flowers were removed from the community. Moreover, two species (*P. fragarioides* and *T. lanceolata* ) among the remaining three flowering plants, which exhibited neutral effects on pollen load when *R. tanguticus* was removed, experienced stronger phenotypic selection on flower size due to increased opportunities for selection. These findings show that the loss of the flowering‐dominant *R. tanguticus* in alpine communities can intensify selection on floral attractive traits of co‐flowering plants, independent of the nature of interspecific interactions. This highlights the evolutionary consequences of changes in community composition and biotic interactions in response to environmental shifts.

## Graphical abstract

This study shows that the loss of the flowering‐dominant R. tanguticus in alpine communities can intensify selection on floral attractive traits of co‐flowering plants, independent of the nature of interspecific interactions. This highlights the evolutionary consequences of changes in community composition and biotic interactions in response to environmental shifts.

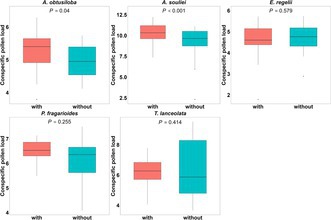

## 1 Introduction

Species interactions are widely recognized as critical in structuring ecological communities. Among these interactions, competition for limited resources plays a key role in regulating species coexistence and maintaining diversity (Grime 1977; Tilman 1982). Plant species compete not only for abiotic resources such as light, nutrients, and water, but also for pollination services (Mitchell et al. 2009). Increasing attention has been directed towards the influence of pollinator sharing in shaping plant communities (Palmer et al. 2003). In particular, competition for pollinator services (exploitative competition) and interference competition through interspecific pollen transfer have been identified as significant forces that shape plant communities (Rathcke 1983; Waser 1983; Campbell 1985). These competitive interactions can influence community structure either through ecological processes, such as species sorting, where inferior competitors are excluded from communities, or through evolutionary mechanisms, such as character displacement, where directional selection drives divergence in traits related to plant–pollinator interactions. Consequently, plant coexistence may be facilitated by partitioning pollinator services through trait divergence, differences in flowering times, pollen presentation schedules, or the placement of pollen on pollinators (e.g., Armbruster et al. 1994).

Fluctuations in the intensity of biotic interactions can significantly influence biotic selection (Benkman 2013; Sletvold and Ågren 2016). When co‐flowering plant species share pollinators, there is an increasing potential for pollinator‐mediated plant–plant interactions (Sargent and Ackerly 2008; Pauw 2013; Carvalheiro et al. 2014). These reproductive interactions can have a profound impact on plant fitness (Arceo‐Gómez and Ashman 2011; Parra‐Tabla and Arceo‐Gómez 2021). The impact of these interactions on plant reproductive performance can vary from positive (facilitation) to neutral or negative (competition) (Morales and Traveset 2008; Hegland et al. 2009; Opedal and Hegland 2020; Bi et al. 2024a). The presence of variation in reproductive success is anticipated to be associated with the opportunities for selection (Benkman 2013; Vanhoenacker et al. 2013). Selection may be diminished if facilitation or reduced competition occurs, which lessens the variation in reproductive success among individuals and consequently the potential for selection (Cardinale et al. 2007; Benkman 2013). On the other hand, selection may be intensified in scenarios where competition enhances the variance in reproductive success, thereby increasing the opportunities for selection (Vamosi et al. 2006; Benkman 2013).

A substantial body of literature on the interaction between co‐occurring plants has investigated whether the richness and density of co‐flowering species influence patterns of selection (see Eisen et al. 2020). Yet the role of key species in an ecological community remains unclear. Dominant species, characterized by their high abundance or biomass within a community, are likely to exert the most significant impact on their neighbors (Smith et al. 2020). A flowering‐dominant species can have either a positive (facilitative) or negative (competitive) effect on the reproductive performance of co‐flowering plants within a community (Morales and Traveset 2008; Hegland et al. 2009; Opedal and Hegland 2020; Parra‐Tabla et al. 2021). For instance, some studies have demonstrated that invasive flowering‐dominant species can diminish the reproductive success of native species (Arceo‐Gómez and Ashman 2011, 2014, 2016; Ye et al. 2014), while others have shown that flowering‐dominant plants can enhance seed set in other community members (Ghazoul 2006; Moreira‐Hernández and Muchhala 2019; Hernández‐Castellano et al. 2020). Experiments involving the removal (or addition) of dominant species from natural communities offer the most robust tests for hypotheses regarding the consequences of reproductive interactions among plant species, including interaction‐mediated phenotypic evolution (Verdú et al. 2021). Specifically, the presence of a dominant competitor for pollination should exert stronger selection on traits of neighboring plants that either enhance attractiveness to increase pollinator visitation or improve mechanical fit to optimize the receipt of conspecific pollen and minimize the receipt of heterospecific pollen (Caruso 1999). However, few studies have been conducted in natural communities to examine the evolutionary consequences of pollinator‐mediated interspecific interactions within ecological communities.

In this study, we conducted a removal experiment within an alpine plant community to investigate the impact of a dominant flowering plant on the fitness components of neighboring species and the variation in patterns of phenotypic selection on floral traits. In the alpine community of the Tibetan Plateau, the overall quantitative effects of pollinator‐mediated interspecific interactions were predominantly positive or neutral (Bi et al. 2024a), in which the dominant flowering plant *Ranunculus tanguticus* acts as a hub‐donor species for pollen transfer. Furthermore, this plant can enhance pollinator visitation of the co‐flowering plants (Bi et al. 2024b). This suggests that the presence of a dominant flowering plant could influence the pollination processes of neighboring plants and potentially alter natural selection on pollination‐associated traits. Consequently, through a manipulative experiment involving the removal of *R. tanguticus* flowers in an alpine community, we aim to examine: (1) the effects of a dominant flowering plant on pollen deposition on the stigma of five neighboring plant species, and whether these interactions are facilitative, neutral, or competitive; (2) whether the removal treatment modifies the pattern of phenotypic selection on floral traits of neighboring plant species. If the interaction is facilitative, we anticipate intensified selection due to weak facilitation when the dominant plant is removed; conversely, for competitive interactions, we expect relaxed selection.

## 2 Materials and Methods

### 2.1 Study Site

This study was conducted in Gansu Gannan Grassland Ecosystem National Observation and Research Station (33°40′ N, 101°52′ E, 3500 m.a.s.l.) in Maqu, Gansu Province, China. The site is characterized by typical alpine meadow vegetation on the Tibetan Plateau, average annual rainfall of about 670 mm, and average annual temperature of 1.2°C. The natural vegetation is dominated by *Kobresia capillifolia*, *Anemone rivulari*, *Carex kansuensis*, *Elymus nutans*, and *Ranunculus tanguticus* (Luo et al. 2006).

### 2.2 Experimental Design and Measurements

In 2019, we established a removal experiment with five 25 × 25 m blocks. Each block contained two 5 × 5 m plots assigned to one of two treatments: the control and the treatment of removing flowers from dominant plants. The plots within each block were spaced 5 m apart, while the blocks themselves were spaced 50 m apart. This arrangement was designed to minimize interference between treatments. In the studied community, *Ranunculus tanguticus* emerged as the dominant flowering plant, with a relative abundance of 0.541 ± 0.166 over a three‐year period. We selected plots with similar density (15–20 individuals per m^2^) of *R. tanguticus* to ensure consistency across our experiment. The removal treatment involved the selective cutting of all flowers from *R. tanguticus* individuals within each plot, while leaving the rest of the plant structures untouched. This approach was designed to maintain the resource competition between *R. tanguticus* and other plant species, but to eliminate the pollination interactions. The focal flowers of *R. tanguticus* were continuously removed from May to July, coinciding with the flowering season.

To access the effects of the removal treatment on other co‐flowering plants within communities, we selected five flowering plant species from the study community (Table 1) and measured their plant height, flower size and stigma position. For each species per plot, we randomly marked five to eight individuals to ensure a representative sample. Plant height were measured using a 50 cm steel ruler. For flower size, we employed Hegland and Totland's method (Hegland and Totland 2005), which categorizes the calculation based on the corolla shape of the plant species. For species with flat, rounded corollas, flower size was calculated as formula *πr* ^2^, where r is the radius of the corolla. For species with papilionaceous corollas, the formula *L × W × l × w* was used, with *L* being Length of the vexil petal, *W* the Width of the vexil petal, *l* the length of the wing petal, *w* the width of the wing petal. For species with tubular and flat corollas, the formula *πr* ^2^ + *πBD* was applied, where *B* is the bore diameter of the tubular structure, and *D* is the corolla tube length. Stigma position was determined by measuring the exsertion of the flower style, which is the length of the style extending beyond the corolla.

**TABLE 1:** Six plant species in this study.

Plant species	Life history	Mating system	Sample size (control, treatment)	Flowering and fruiting periods
*Ranunculus tanguticus*	Perennial	Self‐compatibility	—	Jun–Oct
*Anemone obtusiloba*	Perennial	Self‐compatibility	29, 25	May–Jul
*Aster souliei*	Perennial	Self‐compatibility	20, 20	May–Aug
*Euphrasia regelii*	Annual	Self‐compatibility	25, 25	Jun–Sept
*Potentilla fragarioides*	Perennial	Self‐compatibility	10, 20	May–Aug
*Thermopsis lanceolata*	Perennial	Self‐compatibility	22, 20	May–Aug

To quantify the pollen loads on the stigma, we randomly selected stigmas from one flower in the late stage of anthesis from each marked individual across all plant species. This approach ensures that we capture the pollen load at a critical point in the flower's reproductive cycle. In the laboratory, the stigma of each flower was removed with clean forceps and squashed on a slide with a small cube of fuchsin jelly, then covered with a coverslip. Using a 400× microscope, we counted all the pollen grains present on each stigma. This detailed count included both conspecific (CP) and heterospecific (HP) pollen grains. The distinction between CP and HP was made possible by comparing the size and shape of the pollen grains with a reference library of pollen types from the flowering plants native to the study site, as detailed in Bi et al. (2024a).

### 2.3 Statistical Analysis

We used linear mixed models (LMMs) to evaluate the effects of the removal treatment on each plant species. In these models, the stigmatic pollen load (i.e., conspecific pollen) was treated as the response variable, with the treatment as a fixed factor and block as a random factor. Data were log‐transformed to improve the normality of the residuals.

We applied the methods proposed by Lande and Arnold (1983) to estimate standardized directional selection differentials for each combination of floral trait and conspecific pollen on stigma, utilizing a series of simple linear regression models. The selection differentials include both direct selection on a trait and indirect selection on the trait via correlated characters, representing net selection on a trait. Conspecific pollen on stigma was relativized with the population mean fitness, and plant traits were standardized to make the mean trait value of the whole population equal zero and variance equal one. We then used these standardized traits as independent variables and relative fitness as the dependent variable in our linear regression models. The slope of each regression line corresponds to the directional phenotypic selection differential for the respective trait. Additionally, we fitted an ANCOVA model with each standardized trait, treatment, and trait × treatment interaction as independent variables, with relative conspecific pollen as the dependent variable. A significant trait × treatment interaction indicates a change in phenotypic selection differentials. To check for multicollinearity, we calculated the variance inflation factors (VIF) for all models, which were all below 4, indicating no severe multicollinearity issues. We also quantified the opportunity for selection as the variance in relative fitness for conspecific pollen load for each species. We examined the differences in selection opportunity between treatments using one‐way ANOVA. The data met the assumption of normality, and no transformations were applied. All statistical analyses were conducted using R version 4.2.2 (R Core Team 2022).

## 3 Results

**FIGURE 1:**
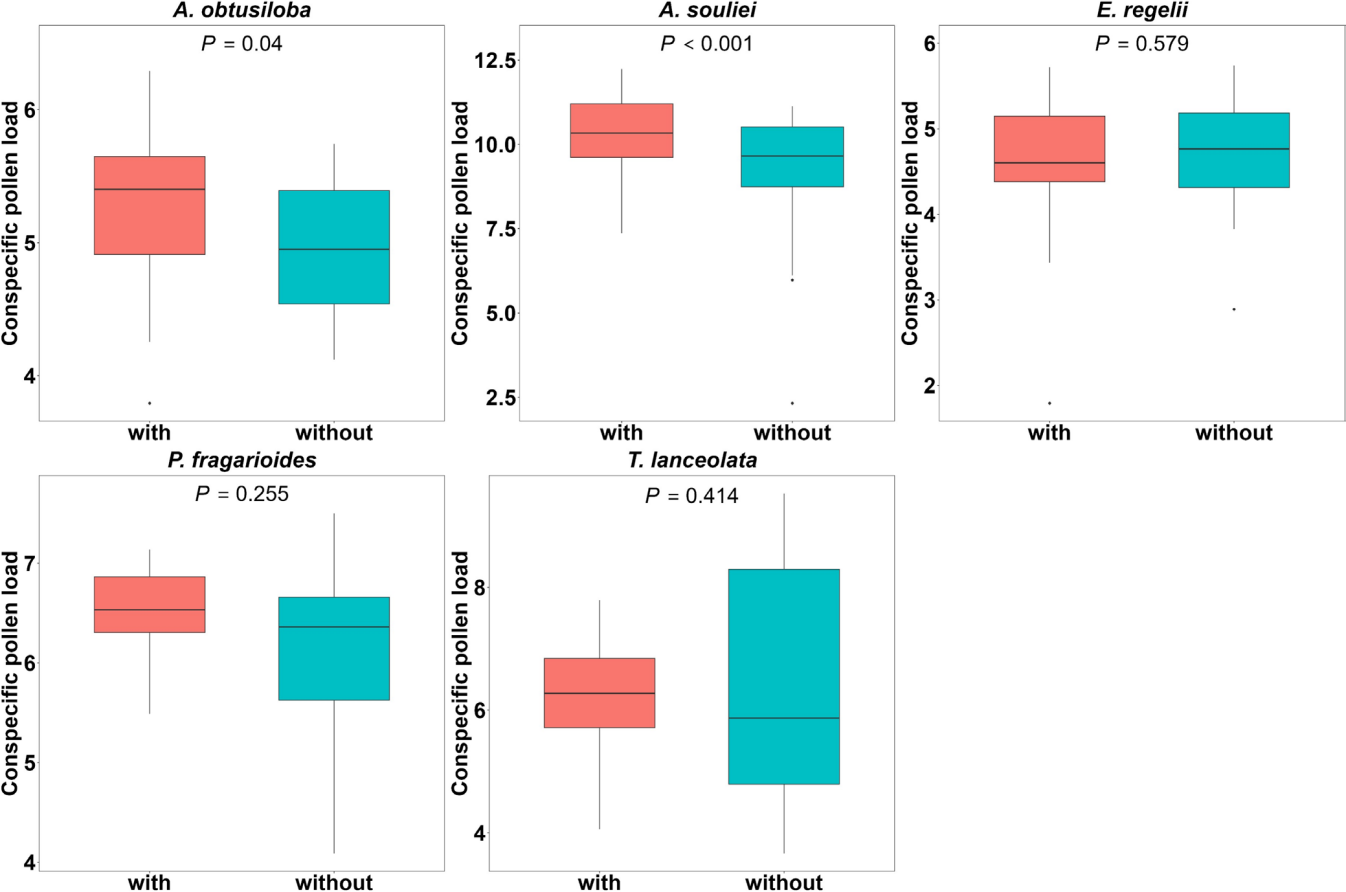
Effects of the removal treatment on conspecific pollen load on stigma for five plant species. (with presence of *Ranunculus tanguticus*, without removing *R. tanguticus* flowers).

**FIGURE 2:**
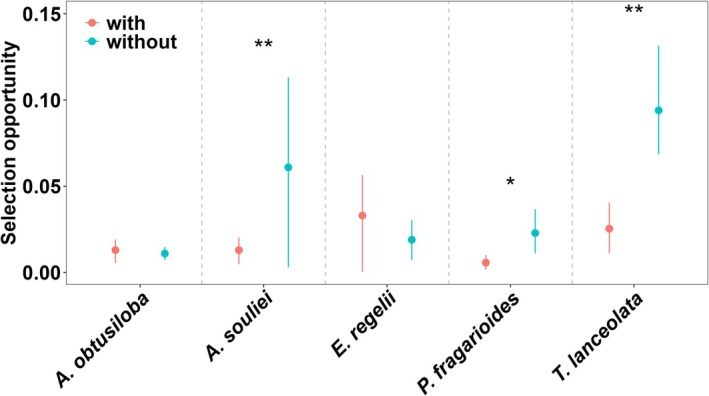
Variation of selection opportunity between treatments for five plant species. “red circle” means the treatment with dominant flowering species of *Ranunculus tanguticus*; “blue circle” means the treatment removing *R. tanguticus* flowers. *p < 0.05, **p < 0.01.

We found a species‐specific effect of the removal of flowering‐dominant plants on the conspecific pollen load on stigma, as indicated by a significant treatment × species interaction (*χ* ^2^ = 14.4207, *p* = 0.0061, Table 2). The deposition of conspecific pollen on stigmas significantly decreased for *A. obtusiloba* and for *A. souliei* in the removal treatment in comparison to the control, suggesting a facilitative effect of *Ranunculus tanguticus*. In contrast, the stigmatic pollen load for the remaining three species showed no variation (Figure 1, Table 3). Furthermore, the removal treatment increased the opportunity for selection based on pollen load for three species: *A. souliei* , *P. fragarioides*, and *T. lanceolata* (Figure 2, Table 4).

**TABLE 2:** Effects of the removal treatment and plant species on conspecific pollen load.

	*χ* ^2^	df	*p*
Treatment	1.0696	1	0.301
Species	333.7864	5	0.001
Treatment × species	14.4027	5	0.0061

**TABLE 3:** Effects of the removal treatment on conspecific pollen load of five neighboring species. *p* values < 0.05 are given in bold.

Species	Estimate	SE	*p*
*A. obtusiloba*	−0.325	0.157	**0.043**
*A. souliei*	−0.169	0.080	**0.041**
*E. regelii*	0.147	0.209	0.486
*P. fragarioides*	−0.409	0.314	0.203
*T. lanceolata*	0.307	0.499	0.541

**TABLE 4:** Opportunity for selection in the control (*I*_C_) and the removal treatment (*I*_T_). Values in parentheses are 95% confidence intervals.

Species	*I*_C_	*I*_T_	*p*
*A. obtusiloba*	0.013 (0.0055, 0.0192)	0.011 (0.0073, 0.0148)	0.7008
*A. souliei*	0.013 (0.0048, 0.0203)	0.061 (0.003, 0.1131)	0.0015
*E. regelii*	0.033 (0.0004, 0.0564)	0.019 (0.0071, 0.0303)	0.2065
*P. fragarioides*	0.0057 (0.0017, 0.0101)	0.0029 (0.0111, 0.0367)	0.039
*T. lanceolata*	0.0254 (0.0111, 0.0405)	0.094 (0.0686, 0.1315)	0.0074

**FIGURE 3:**
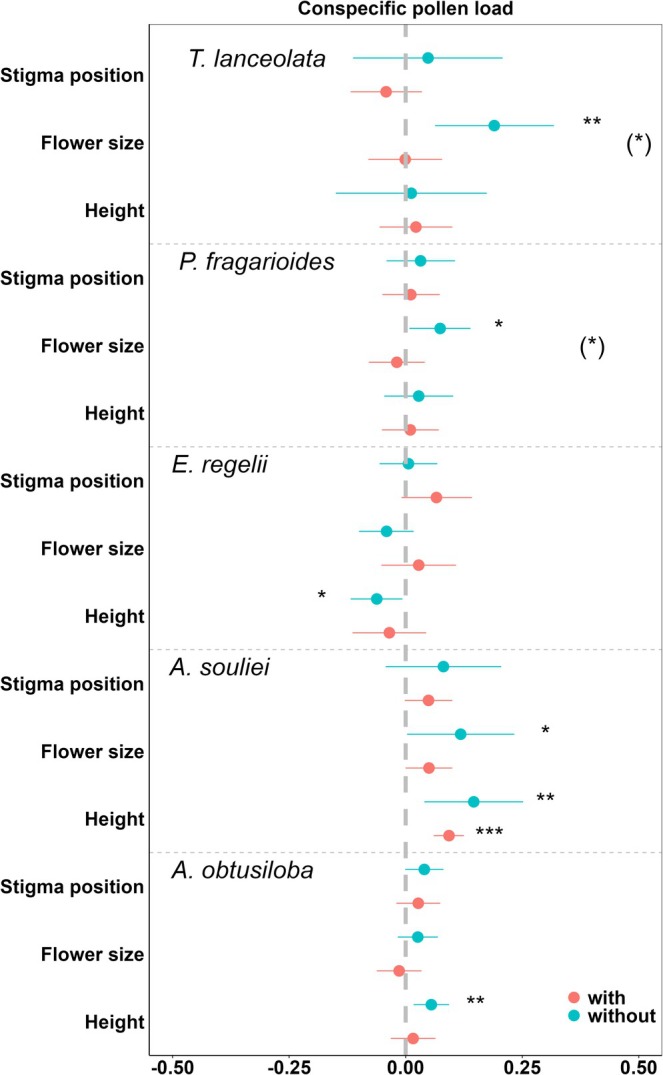
Effect size plot for directional selection differential through conspecific pollen on stigma on three flower traits of five species. Dots denote selection differential and error bars denotes 95% CI. Star (*, **, ***) means significant selection differential where error bars did not overlap zero. (*) means significant trait × treatment interaction. “red circle” means the treatment with dominant flowering species of *Ranunculus tanguticus*; “blue circle” means the treatment removing *R. tanguticus* flowers.

**FIGURE 4:**
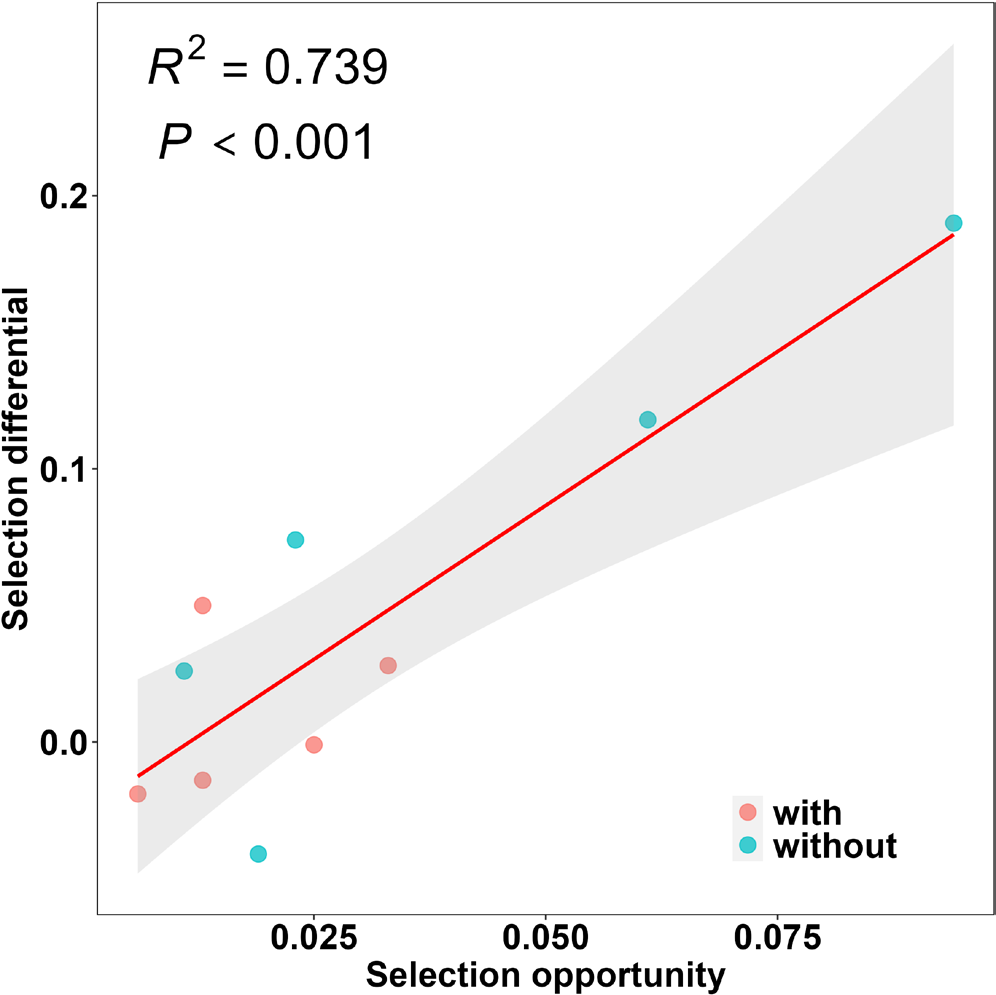
The relationship between net selection (selection differential) on flower size and selection opportunity across the treatments for five plant species. “red circle” means the treatment with dominant flowering species of *Ranunculus tanguticus*; “blue circle” means the treatment removing *R. tanguticus* flowers. The gray area represents 95% confidence interval.

The removal of *R. tanguticus* flowers had distinct effects on the phenotypic selection patterns for five plant species (Figure 3, Table 5). Significant net selections, that is, selection differentials, were more frequently observed in the removal treatment compared to the control. Selection differentials for flower size were significant for *A. souliei* , *P. fragarioides*, and *T. lanceolata* in the removal treatment, with the latter two species showing significant differences between treatments. For plant height, selection differentials were significant for *E. regelii* and *A. obtusiloba* in the removal treatment and for *A. souliei* in both treatments. No significant net selection on stigma position was found across all five species. Additionally, the selection differential on flower size was positively correlated with selection opportunity in all studied species (Figure 4).

**TABLE 5:** Variance‐standardized univariate selection differentials in the control (*β*_C_) and the removal treatment (*β*_T_) for five species. Values in parentheses are 95% confidence intervals. Bold text indicates statistically significant estimates (< 0.05).

Species	Traits	*β*_C_	*β*_T_	Trait × treatment, *p*
*A. obtusiloba*	Plant height (cm)	0.016 (−0.032, 0.064)	**0.055 (0.017, 0.093)**	0.192
	Flower size	−0.014 (−0.062, 0.034)	0.026 (−0.017, 0.069)	0.206
	Stigma position (mm)	0.027 (−0.02, 0.074)	0.04 (−0.001, 0.081)	0.671
*A. souliei*	Plant height (cm)	**0.093 (0.06, 0.125)**	**0.146 (0.04, 0.252)**	0.313
	Flower size	**0.05 (−0.0002, 0.1)**	**0.118 (0.003, 0.233)**	0.262
	Stigma position (mm)	0.049 (−0.002, 0.1)	0.081 (−0.043, 0.205)	0.619
*E. regelii*	Plant height (cm)	−0.0035 (−0.114, 0.044)	**−0.062 (−0.118, −0.007)**	0.564
	Flower size	0.028 (−0.052, 0.108)	−0.041 (−0.1, 0.017)	0.156
	Stigma position (mm)	0.066 (−0.009, 0.142)	0.006 (−0.056, 0.068)	0.205
*P. fragarioides*	Plant height (cm)	**0.01 (−0.051, 0.071)**	0.028 (−0.046, 0.102)	0.697
	Flower size	−0.019 (−0.079, 0.041)	**0.074 (0.008, 0.139)**	**0.032**
	Stigma position (mm)	0.011 (−0.05, 0.073)	0.032 (−0.041, 0.106)	0.636
*T. lanceolata*	Plant height (cm)	0.022 (−0.056, 0.1)	0.012 (−0.15, 0.174)	0.907
	Flower size	−0.001 (−0.08, 0.078)	**0.19 (0.063, 0.318)**	**0.011**
	Stigma position (mm)	−0.042 (−0.118, 0.035)	0.048 (−0.113, 0.208)	0.264

*Note:* The bold values indicate that the selection differential is significant (*p* < 0.05) in the treatment.

## 4 Discussion

This study found that the removal of flowers from a dominant flowering plant, *Ranunculus tanguticus*, in alpine grasslands had different effects on the pollen deposition of neighboring flowering plants and altered the pattern of pollinator‐mediated selection on floral traits. *R. tanguticus* had a positive effect on the pollen receipt of two plant species, *A. obtusiloba* and *A. souliei* . Correspondingly, flower‐attractive traits (flower height and size) rather than the mechanical‐fit trait (stigma position) of *A. obtusiloba* and *A. souliei* , were subjected to significant net selection (i.e., selection differential) when *R. tanguticus* flowers were removed from the community. Moreover, two species (*P. fragarioides* and *T. lanceolata* ) among the other three flowering plants, which exhibited neutral effects on pollen load when *R. tanguticus* was removed, experienced stronger phenotypic selection on flower size due to increased opportunities for selection. These results show that the loss of the flowering‐dominant *R. tanguticus* in alpine communities can intensify selection on floral‐attractive traits of co‐flowering plants, independent of the sign of interspecific interactions.

As we predicted, interspecific facilitative interactions have been shown to weaken the phenotypic selection on flower traits among neighboring flowering plants. Our previous study indicated that the flowering‐dominant species *R. tanguticus* enhances pollinator visitation and seed set for neighboring plants within alpine communities (Bi et al. 2024b). Building on these findings, our current study reveals that this species exerts a facilitative influence on pollen receipt for two co‐flowering plants, *A. obtusiloba* and *A. souliei* , by sharing pollinators. In populations of animal‐pollinated plants, variation in pollination success among individuals arises when pollinator visitation is low and fecundity is pollen‐limited, leading to pollinator‐mediated selection if trait expression affects the probability of pollen transfer. Conversely, when pollinator visitation is high and most plants are saturated with compatible pollen, the potential for selection through differential pollination success is reduced. Pollinator‐mediated selection is therefore weaker when pollination is reliable (with little or no pollen limitation) and stronger and more variable when pollination is unreliable (Ashman and Morgan 2004; Bartkowska and Johnston 2015; Sletvold and Ågren 2016; Trunschke et al. 2017; Albertsen et al. 2021). Given the facilitative effect of *R. tanguticus*, its loss could increase pollination limitation for neighboring plants, thereby intensifying selection on flower size and height for *A. obtusiloba* and *A. souliei* . This intensified selection suggests that changes in flower size have a more significant impact on individual fitness when these focal species are not surrounded by *R. tanguticus*. In this study, phenotypic selection is pollinator‐mediated, as relative fitness was estimated based on the number of conspecific pollen grains on stigmas, indicating that pollinators are the agents of selection. Furthermore, this experiment eliminated confounding effects of environmental factors and specifically assessed the role of flowers in mediating pollination interactions without altering non‐reproductive plant interactions. Our results align with the general pattern that facilitation or reduced competition can lead to weaker selection in more species‐rich communities (Moeller and Geber 2005; Wassink and Caruso 2013; Parachnowitsch et al. 2014; Eisen et al. 2020). For example, Moeller and Geber (2005) found that the presence of pollinator‐sharing congeners in the experimental populations increased bee visitation to *Clarkia xantiana* and reduced the strength of phenotypic selection on mating system traits. While studies have demonstrated that interspecific facilitation with a greater number of species can weaken phenotypic selection on flower traits (see Eisen et al. 2020), few have examined whether pollination facilitation of individual flowering plants can alleviate natural selection mediated by pollinators in ecological communities. Our findings demonstrate that pollinator‐mediated interspecific interactions play a significant role in shaping the patterns of phenotypic selection and driving the evolution of floral traits within multi‐species communities. This influence is particularly pronounced in communities undergoing transformations due to environmental changes.

Interactions between neighboring plant species, which can range from facilitative to competitive, have been documented across various ecosystems (e.g., Moragues and Traveset 2005; Muñoz and Cavieres 2008; Seifan et al. 2014; Eisen et al. 2020). In alpine meadows, Bi et al. (2024a) observed less pollination competition among co‐flowering plants, where the overall quantitative effects of pollinator‐mediated interspecific interactions were predominantly positive (facilitative) or neutral (with no detectable effect). In addition to identifying a facilitative effect on two plant species ( *A. obtusiloba* and *A. souliei* ), we also found that the reproductive interaction between the dominant *R. tanguticus* and three other co‐flowering plants (*E. regelii*, *P. fragarioides*, and *T. lanceolata* ) was neutral. In our prior research, we discovered that the presence of *R. tanguticus* flowers augmented pollinator visits to *A. souliei* as opposed to *A. obtusiloba* (Bi et al. 2024b). This difference in reproductive interactions across plant species could be attributed to variations in community composition and floral trait similarity. Specifically, more similar plant species are likely to share pollinators more effectively for pollen transfer, which may be one of the key mechanisms underlying interspecific facilitation in high‐altitude habitats (Bi et al. 2024b). However, when *R. tanguticus* was absent, phenotypic selection differentials, as estimated by pollen deposition, favored larger flowers in two plant species, *P. fragarioides* and *T. lanceolata* . Similarly, Wassink and Caruso (2013) conducted an artificial experiment cultivating *Lobelia siphilitica* L. in the presence and absence of *Mimulus ringens* . They demonstrated that *M. ringens* did not influence the seed set of *L. siphilitica* , yet there was significant selection for smaller daily displays in the absence of *M. ringens* , and nonsignificant selection for larger displays in its presence. In our study, the removal of *R. tanguticus* increased the selection opportunity based on pollen load for three species: *A. souliei* , *P. fragarioides*, and *T. lanceolata* (Figure 2). This increase was characterized by a larger variation in pollen receipt among individuals without *R. tanguticus* flowers in the communities. The opportunity for selection in this study was positively correlated with net selection on flower size (Figure 3), indicating that variation in reproductive success is linked to the opportunity for selection and can lead to stronger selection in treatments where *R. tanguticus* is absent (Benkman 2013; Vanhoenacker et al. 2013).

In conclusion, this study demonstrates that the reproductive interactions between the flowering‐dominant *R. tanguticus* and its co‐flowering neighbors in alpine meadows are either positive or neutral. The presence of *R. tanguticus* flowers mitigates pollinator‐mediated selection on floral attractive traits, which are attributed to decreased opportunities for selection, regardless of interspecific type. By experimentally removing the dominant plant, this study reveals for the first time the indirect pollinator‐mediated selection on floral traits of neighboring plants within multi‐species flowering communities. This finding highlights the role of keystone species, such as dominant *R. tanguticus*, in regulating community‐level evolutionary trajectories. Long‐term monitoring could further elucidate whether current neutral/positive interactions transition to competition in response to changes in community composition induced by environmental shifts. Our work also provides a functional criterion for identifying keystone species in alpine meadows, emphasizing the need to prioritize their protection in conservation strategies.

## Author Contributions

**Yan Ma:** formal analysis (equal), writing – original draft (equal). **Xiaoli Wang:** writing – original draft (equal). **Yizhi Qiu:** formal analysis (equal). **Zhigang Zhao:** funding acquisition (equal), supervision (equal), writing – original draft (equal).

## Conflicts of Interest

The authors declare no conflicts of interest.

## Data Availability

The data that support the findings of this study are openly available in “figshare” at http://doi.org/10.6084/m9.figshare.27959130.
